# Naïve hosts of avian brood parasites accept foreign eggs, whereas older hosts fine-tune foreign egg discrimination during laying

**DOI:** 10.1186/1742-9994-11-45

**Published:** 2014-06-27

**Authors:** Csaba Moskát, Miklós Bán, Márk E Hauber

**Affiliations:** 1 MTA-ELTE-MTM Ecology Research Group, c/o Biological Institute, Eötvös Lóránd University, Pázmány Péter sétány 1/C., H-1117 Budapest, Hungary and Hungarian Natural History Museum, Baross u. 13, Budapest H-1088, Hungary; 2 MTA-DE “Lendület” Behavioural Ecology Research Group, Department of Evolutionary Zoology, University of Debrecen, Debrecen 4010, Hungary; 3 Department of Psychology, Hunter College and the Graduate Center of the City University of New York, 695 Park Avenue, New York, NY 10065, USA

**Keywords:** Adaptation, Brood parasitism, Clutch learning, Egg discrimination

## Abstract

**Background:**

Many potential hosts of social parasites recognize and reject foreign intruders, and reduce or altogether escape the negative impacts of parasitism. The ontogenetic basis of whether and how avian hosts recognize their own and the brood parasitic eggs remains unclear. By repeatedly parasitizing the same hosts with a consistent parasitic egg type, and contrasting the responses of naïve and older breeders, we studied ontogenetic plasticity in the rejection of foreign eggs by the great reed warbler (*Acrocephalus arundinaceus*), a host species of the common cuckoo (*Cuculus canorus*).

**Results:**

In response to experimental parasitism before the onset of laying, first time breeding hosts showed almost no egg ejection, compared to higher rates of ejection in older breeders. Young birds continued to accept foreign eggs when they were subjected to repeated parasitism, whereas older birds showed even higher ejection rates later in the same laying cycle.

**Conclusions:**

Our results are consistent with the hypotheses that (i) naïve hosts need to see and learn the appearance of their own eggs to discriminate and reject foreign eggs, whereas (ii) experienced breeders possess a recognition template of their own eggs and reject parasitic eggs even without having to see their own eggs. However, we cannot exclude the possibility that other external cues and internal processes, accumulated simply with increasing age, may also modify age-specific patterns in egg rejection (e.g. more sightings of the cuckoo by older breeders). Future research should specifically track the potential role of learning in responses of individual hosts between first and subsequent breeding attempts by testing whether imprinting on a parasitized clutch reduces the rates of rejecting foreign eggs in subsequent parasitized clutches.

## Introduction

The visual, auditory or olfactory recognition of enemies, including predators by their prey or brood parasites by their hosts, and the subsequent avoidance of the predators’ vicinity or the rejection of parasites’ offspring, are critical for fitness. But how do naïve individuals of potential prey or host species recognize their natural enemies? There are diverse feasible cognitive mechanisms of such recognition, and its ontogeny may include innate templates or learned cues (e.g. [1]). For example, when prey are exposed to and learn about predators earlier in life, they may use specific cues towards establishing a broader template inclusive of other predator species, thus increasing the accuracy and reducing the latency of later behavioural decisions to escape. Here we consider avian host-brood parasite recognition systems, which have become a well-established model for the study of co-evolutionary arms races [2]. We focus on the role of age-dependence in general, and the host’s learning of its own eggs’ appearance in particular, because a critical role for learning by naïve breeders, to recognize their own eggs and to reject parasitic eggs, has been repeatedly suggested by different theoretical [3,4] and empirical studies [5,6].

**Figure 1:**
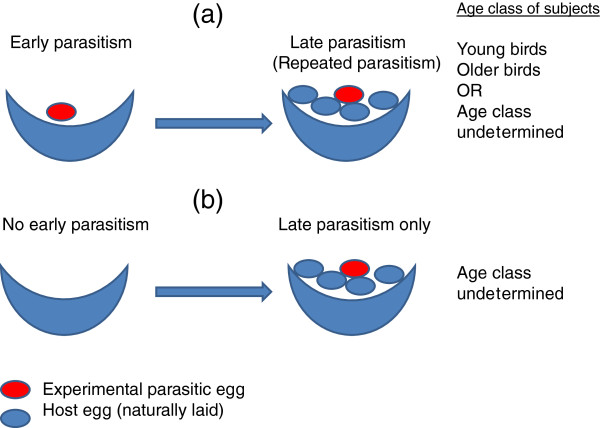
**Schematic representation of the nest manipulation treatments applied in the study, with reference to age classification.** In “Early parasitism” an experimental parasitic egg was placed into a complete but empty nest, and monitored for ejection, desertion or acceptance (and removed if still present on the day before “Late parasitism” started); in “Late parasitism” one host egg was exchanged with an experimental parasitic egg on the day when the natural clutch size reached 5 eggs and the nest was monitored for ejection, desertion or acceptance for 5 days.

Interspecific brood parasitism is a widespread phenomenon in diverse lineages, including birds [2,7], fishes [8]), arachnids [9,10] and social insects [11-13]. Amongst avian brood parasites and their hosts, learning to recognize and reject the parasite’s egg is an important step in the co-evolutionary arms race [14], whereby the host defends itself from the parasite through lowering or eliminating the costs of raising parasitic offspring [12,15]. Here we assessed the role of ontogeny and breeding experience in explaining variation of the egg rejection behaviours of the host of an obligate avian brood parasite, at two temporal scales: between early and late stages of a single laying cycle, and between young, naïve breeders and older, presumably experienced breeders (Figure 1).

Previous empirical work, mostly in birds and insects, showed that some young (naïve) hosts are less effective in the rejection of parasitic offspring compared to older and more experienced hosts, suggesting a role for learned components in antiparasitic defenses [2,16]. For example, in some species, more experienced avian hosts are better than naïve hosts at recognizing the same parasitic species’ eggs or nestlings [5,17,18], but see [19-22]. In this study, we assessed the role of an avian host’s age, and presumed prior breeding experience, to directly test for ontogenetic predictors of egg ejection plasticity in young vs. older hosts in response to single or multiple parasitism in the same egg-laying cycle.

Social learning plays a role in the defence behaviours of some potential hosts: they can learn to identify adult brood parasites, and to protect their nest by mobbing nearby parasites that resemble those parasites which they had previously witnessed neighbouring hosts to mob [23,24]. On the one hand, in egg discrimination potential hosts cannot reliably observe how others recognize and reject the foreign egg already inside the nest, thereby eliminating social learning altogether in the ontogeny of egg recognition, and narrowing the basis of its developmental plasticity to an individual’s own experiences with eggs in the nest [25], but see [26]. On the other hand, the presence of adult common cuckoos (*Cuculus canorus*) at the nests may increase the frequency of egg rejection, as seen in the great reed warbler (*Acrocephalus arundinaceus*), but only when the mimicry of the foreign eggs is poor [27]. Published work on the closely related oriental reed warbler (*Acrocephalus orientalis*) in Japan suggested that older hosts had more accurate abilities than naïve breeders to reject natural cuckoo eggs [5]. However, a handful of follow-up studies failed to reveal such age-dependency of the rejection of the parasite’s eggs in other cuckoo host species [20-22,28]. Therefore, it remains unclear whether and how hosts learn from their earliest breeding experiences to recognize parasitic eggs, and whether this learning carries the cost of misimprinting on foreign eggs so that in future nesting attempt the host would respond to cuckoo eggs as it would to its own eggs.

The great reed warbler shows extensive variation in the probability with which it rejects natural common cuckoo eggs (~33% as an average) [29] or experimental foreign eggs (0-100%) introduced into the nest [30,31]. This host is known to use one of several, or a combination of different cognitive decision rules, to recognize and reject even mimetic parasitic eggs [31-33]. For example, this species does not need to see its own eggs to reject poorly mimetic foreign eggs from its nest when all host eggs have been experimentally replaced, implying the use of a mimicry-dependent recognition template in responding to different eggs in the nest [31]. In addition, prior exposure to parasitism, whether through adult parasites near the nest [27], or through a non-mimetic foreign egg in the nest [34], yields an increase in the rejection rate of moderately mimetic foreign eggs in the nest, implying the use of a shifting acceptance threshold in egg-rejection decisions. Finally, in great reed warblers, mimicry may combine with a discordancy-based egg rejection rule, causing the rejection of even a single, mimetic experimental egg in the clutch (through discordancy), but only when it is in the minority of otherwise poorly-mimetic eggs in the majority of the clutch (mimicry-dependence [35]).

To further our understanding about the developmental and cognitive mechanisms of egg rejection in the great reed warbler, this study tested ontogenetic explanations of the variation in the rejection of natural and experimental foreign eggs in the nest. Specifically, we assessed whether experimental brood parasitism prior to the onset of laying may modify individuals’ subsequent egg rejection decisions in the latter stages of the same laying cycle when exposed to a second, repeated parasitism event. Following [5], we hypothesized that hosts would learn, and be more likely to accept a foreign egg type if they had seen it as the first egg in their nest. Accordingly, we predicted a low rejection rate of that same egg type in a consecutive, repeated parasitism event, compared to hosts that did not have prior experience with a parasitic egg in the same laying cycle. In contrast, for more experienced breeders, we assumed that most hosts would have not been exposed to early parasitism during their first year of breeding, thereby establishing a recognition template for their own eggs that did *not* include cuckoo eggs. For these hosts, early exposure and rejection of a cuckoo egg during subsequent breeding bouts may facilitate fine-tuning of the recognition template through narrowing their acceptance thresholds for any subsequently laid cuckoo eggs during the same laying cycle [34]. Accordingly, these hosts are predicted to detect and reject more foreign eggs from the nest in subsequent, repeated parasitism, compared to both naïve hosts and to experienced hosts with a single exposure to parasitism. Alternatively, all hosts including naïve breeders may use an existing template of their own eggs’ phenotypes [33]; this latter hypothesis predicts that hosts across all age classes uniformly reject both early- and late-laid parasitic eggs whether in single or in repeated parasitism, irrespective of the timing of parasitism relative to the laying cycle.

## Results

**Figure 2:**
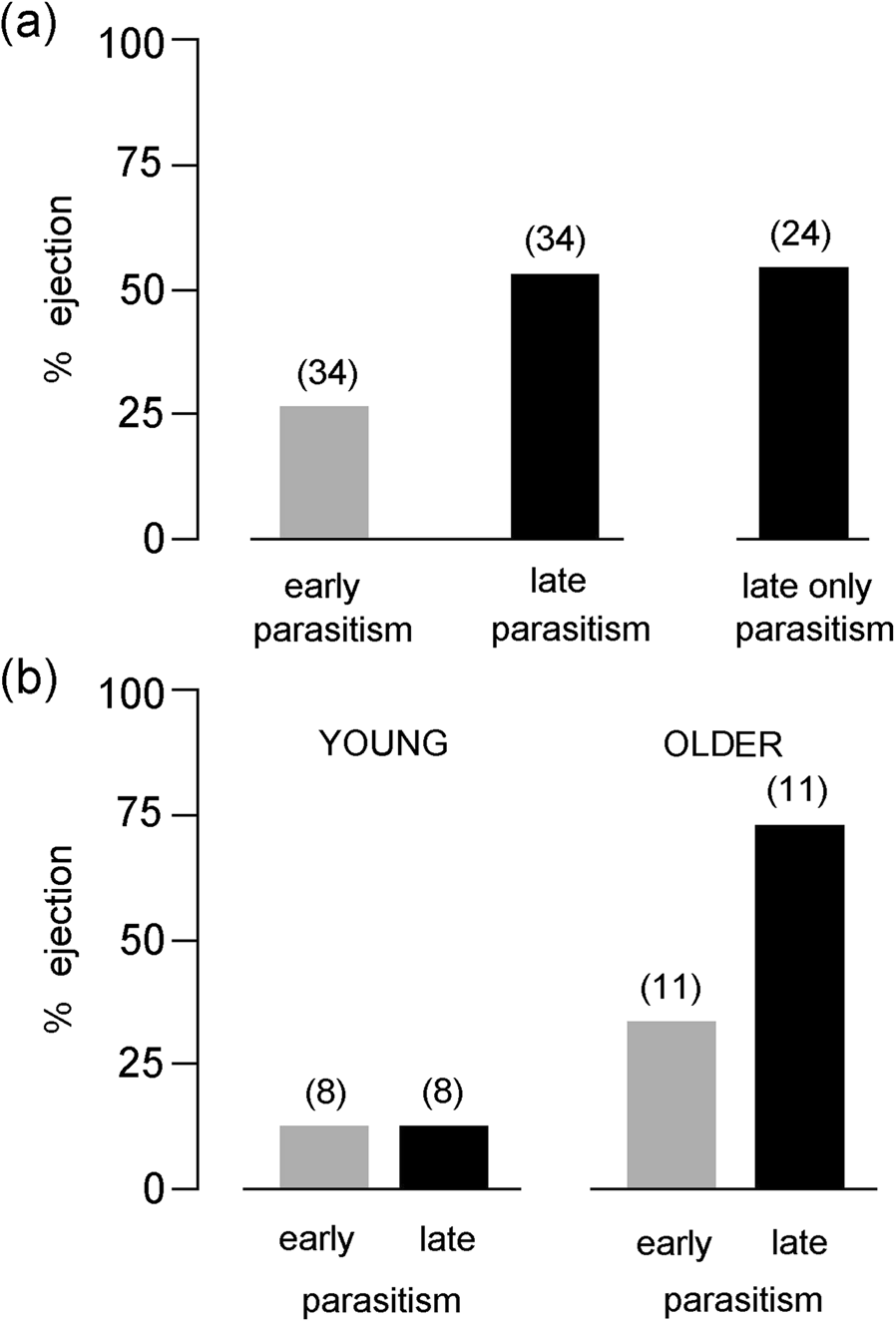
Ejection rates of experimental eggs following single parasitism, or a repeated treatment of two consecutive single parasitisms in the same nest; all nests combined (a); data from nests with known age-classes of females (b).

We completed 34 experiments with the two consecutive, repeated parasitism treatments (“early” and “late”) and 24 experiments with “late-only” treatment (Figure 2). Pooled across all experimental individuals, the ejection rate in “early parasitism” (9/34) was statistically lower overall than in “late parasitism” (18/34) at the same nests (McNemar test, n = 34, p = 0.004) (Figure 2a). Specifically, the ejection rate in “early parasitism” was also lower than in “late-only parasitism” (13/24) (χ^2^_1_ = 4.584, p = 0.032). This is consistent with our previous work which showed that across a population of this same host species with unknown aged individuals, egg rejection responses to natural cuckoo parasitism were lowest when the cuckoo egg is laid prior to the onset of host laying and higher after the hosts have begun egg laying [15].

With these data pooled across all subjects and un/known-age groups, prior exposure to the parasitic egg in the “early” stage, overall, had no effect on hosts’ egg discrimination, as ejection rates were not statistically different in the “late” and “late-only” treatments (χ^2^_1_ = 0.009, p = 0.927). Latencies of egg ejections in early (mean = 2.67 days ± 0.69 s.e.) and late parasitism (mean = 1.67 days ± 0.33 s.e.) also did not differ significantly (Mann–Whitney U_9,18_ = 61, p = 0.322). Although there was no treatment in this study for ‘early parasitism with a prolonged (more than 5 days) monitoring period but without a second trial’, we have data for this comparison from natural cases of cuckoo parasitism from the same study site and year: we found six naturally parasitized nests in the pre-egg laying stage, i.e. empty nests, where hosts ejected the natural cuckoo eggs, where mean latency was 2.33 days (±0.56 s.e.), range: 1–4 days. This value from early natural parasitism did not differ from latencies in our early parasitism experiments (Mann–Whitney U_6,9_ = 24.1, p = 0.776). No host eggs were rejected from unparasitized, control nests (n = 27).

Regarding hosts with known ages, most (88%) known first-time breeders (young hosts, n = 8) were acceptors in both treatments, as only one young female ejected parasitic eggs, doing so in both the “early” and “late” treatments (Figure 2b). In contrast, older hosts (n = 11) showed more rejection overall than did younger hosts (F_19_ = 5.978, p = 0.024), and, as predicted, the older birds’ ejection rate also increased from the “early” to “late” treatments (F_19_ = 4.571, p = 0.046). Overall, we detected a significant interaction of age and the timing of the treatments (F_19_ = 4.571, p = 0.046).

Regarding the repeatability of egg ejection behaviour e.g. [36-40], most hosts responded consistently to parasitism for the two consecutive treatments in the same nests, but, four older hosts changed from acceptors to rejecters and no bird changed from rejecter to acceptor (repeatability of ejection behaviour in young hosts: Spearman’s r = 1.0, n = 8, p < 0.001, exact 95% CI = 1.0-1.0; in older hosts: Spearman’s r = 0.463, n = 11, p = 0.152, exact 95% CI = 0.156-0.770).

## Discussion

Our results on the egg ejection behaviours of great reed warblers, regarding age-specific responses to experimental brood parasitism, support but do not directly test the hypothesis that because older birds have already learned their own eggs’ phenotypes, they can reject more foreign eggs compared to naïve breeders [5,41]. Our experimental data of repeated parasitism across the host laying cycle, however, also highlight the complexity and the interaction of the roles that learning about own vs. cuckoo eggs, and/or experiences accumulated and cognitive-physiological changes occurring with the passage of time (i.e. age) may play in a host’s likelihood to reject foreign eggs from the nest. Specifically, we showed (i) age-dependent effects on egg discrimination: young birds showed significantly lower rates of rejection than older birds in the repeated parasitism treatment, and only older birds showed an increase in egg rejection rates between early and repeated experimental parasitism; together, these patterns reflect an age-specific component of prior experience with parasitism (see also [5]). In turn, these data are consistent with the hypothesis that (ii) a host’s experience with its own eggs in a prior breeding season may lead to a fine-tuning of the rejection of foreign eggs across subsequent laying cycles. Whether older hosts inspect each freshly laid egg to update their recognition template during egg laying in each breeding bout, or use a discordancy-based discrimination mechanism by comparing differences between all eggs in the clutch [35] to detect and reject the foreign egg (of the minority phenotype) at clutch completion [1], remains to be discovered. Additionally, to test theories of egg-(mis) imprinting [42], future studies should also explore whether young, naïve hosts would reject foreign eggs when exposed to these same egg types at the end of their first laying cycle only, and whether naïve hosts exposed to experimental eggs before laying incorporate that egg’s phenotype into their recognition template and continue to accept it in subsequent breeding bouts and seasons, as predicted by Lotem’s theory [42].

Inspection and fine-tuning of egg discrimination at consecutive nesting cycles is critical for great reed warblers, because the appearance of individual host’s eggs in this frequent cuckoo-host species, shows extensive intraseasonal and interannual variation [43] and because the cuckoos preferentially match the appearance of their own eggs with the host eggs’ phenotypes in parasitized nests [44-46]. Such an ongoing assessment process at each laying cycle fine-tunes egg recognition to yield the pattern that, overall, older oriental and great reed warbler hosts of common cuckoos are better at ejecting experimentally introduced parasitic eggs in the nest ( [5]; this study).

One hypothesis predicts that hosts may learn to identify their own eggs based on the first egg they lay at each nesting attempt; thus, all those hosts that are parasitized early, would always accept the parasite’s eggs in later breeding bouts [6,47,48]. Prior results from our host population did not support this clutch learning hypothesis, as swift manipulation of newly-laid host eggs with extra maculation did not lead to the learning of the new, modified egg phenotype, and the hosts continued to reject these modified egg types to the same extent as hosts in nest without daily manipulation of newly laid eggs during the egg laying cycle [33]. However, the differences between that study and the current results may be due to manipulating maculation (prior study) vs. background colour of the egg (this study), which may provide alternative or complementary recognition cues about foreign eggs in the nest [30,49]. Alternatively, other work within the same population also revealed that the successful ejection of the poorly mimetic parasitic egg may help the host recognize a more mimetic parasitic egg at the next instance of repeated parasitism [34], implying a critical role of experience during the laying cycle in modulating egg rejection accuracy and patterns.

Our study also has an important implication for the methodology of egg rejection studies. Previous published studies have completed egg rejection experiments at over 10,000 nests of multiple species [50], nearly all with the method of a single experimental parasitism event with a single foreign egg type, typically at clutch completion, or just before it. However, that method is not suitable for revealing how hosts’ egg discrimination is modulated during the early nesting and egg laying stages (also see [32]). That traditional approach may mask important details, including that older birds increase their knowledge of their own eggs during each egg laying process, which can only be shown by repeated parasitism of the same clutch at the early and late egg-laying stages. Such a methodological difference may also explain why we detected an age-effect in egg rejection behaviours in our study but our colleagues working with the same host species north of us, did not [28]. As a consequence of the population-wide pattern that early-parasitized hosts are less likely to reject parasitic eggs compared to late parasitism (Figure 2), cuckoos may benefit from early parasitism when it is timed around the onset of egg laying, especially when parasitizing first-time breeders, naïve, young hosts. Our experimental protocol followed the pattern seen in natural parasitism at our study site, as great reed warblers are often parasitized twice during their egg laying, with the first time typically just before onset of laying see (Material and methods). The benefits of early parasitism of hosts within the laying cycle may also have important implications for host-parasite mismatch in migratory timing, due to climate change [51,52].

Theoretical models on host egg discrimination have focused on egg-learning by naïve hosts [3,4], but our new results suggest the possibility for extending the generalized model mechanism of egg learning to include age-dependent fine-tuning (e.g. [48]). The main conclusion from our results is that hosts do not follow a simple decision rule for egg rejection based solely on either sensory discrimination [53] or egg- or clutch learning [48]. There is increasing evidence for the role of context-dependence for social recognition mechanisms in avian [18,54] and invertebrate hosts of social parasites [55,56]; our results here provide new evidence to expand the scope of this context-dependence to internal, age-specific effects. On the one hand, age-related egg discrimination followed the theoretically predicted and empirically observed pattern for one other cuckoo host species [5]: naïve hosts were mostly acceptors, whereas more experienced hosts were rejecters. On the other hand, seasonally and annually variable eggshell appearance of great reed warbler clutches in successive breeding attempts [43] sets up the need for ongoing egg learning by these hosts at each breeding attempt. Further research on known-age hosts of cuckoos and other avian brood parasites should clarify whether fine-tuned learning of own eggs during egg-laying is necessary for accurate egg recognition at each nesting attempt.

## Conclusions

We studied the age-dependence of egg rejection behaviours in the great reed warbler, a frequently and repeatedly parasitized host of the brood parasitic common cuckoo. We revealed two main results: (i) naïve, first-time breeders rarely rejected foreign eggs, whereas older, experienced breeders rejected foreign eggs more often; and (ii) older hosts, presumed to have already seen their own eggs in previous year(s) and breeding attempt(s), increased their egg rejection rates across the egg-laying cycle. These findings imply that older hosts can fine-tune their egg recognition during laying, and suggests the use of learning of the variable egg phenotypes laid by the same hosts in each of their breeding attempts. However, we cannot exclude the possibility that other external cues and internal processes, accumulated simply with increasing age, may also modify age-specific patterns in egg rejection (e.g. more sightings of the cuckoo by older breeders).

## Materials and methods

The study was conducted in central Hungary, near Apaj (47°07′N; 19°06′E), where nests of great reed warblers were systematically sought in 2–4 m wide reed-beds (*Phragmites australis*) along small irrigation channels, once or twice a week, from mid-May until mid-June, 2010–2013. This area was heavily parasitized by common cuckoos (ca. 64% of nests; [29]), but for experimentation we used naturally non-parasitized nests. As cuckoos typically parasitize great reed warbler nests within 50 m of their vantage points (trees, electric poles or wires; [57]), to reduce natural cuckoo parasitism during the experiments, we tended to select nests in treeless sections of channels or farther away from trees (>50 m). In our study area about half of the cuckoo eggs were found in multiply-parasitized nests, i.e. the nest contained two or more cuckoo eggs (23% and 13%, respectively [29]). Although both monogamy and polygamy occur among great reed warblers, the mating status of each member of a pair has no effect on parasitism rate [58].

We determined host age by capturing them in mist-nets at about half of the monitored nests, placing a stuffed cuckoo nearby to attract the birds to the proximity of mist-nets. To avoid the potential effect of cuckoo-attacks against stuffed models to increase the hosts’ egg rejection rate [27], we caught nesting birds when the experimental treatments had already concluded. We used a direct measure (combining the coloration of iris, tarsus and tongue spots), rather than an indirect measure (laying date; [59]) as a predictor of age in our study population. In this host, only females incubate and are responsible for egg rejection [60], so we assessed their age categories following the method of [61], based on [62], as second year (young, first time breeder) or after second year (older breeder).

### Early parasitism treatment

We placed a conspecific egg, collected from abandoned clutches, into a complete but empty nest of the host. This egg was painted with a yellow Stabilo Boss highlighter (No. 70/24) pen (hereafter: the parasitic egg). We then inspected nest contents daily. Typically, hosts started egg laying in the next day(s) (range: 1–5 days, mean = 1.706 ± 0.166 s.e.), and the clutch grew daily with a new host egg. When the clutch contained four unmanipulated host eggs, we took out the parasitic egg from the nest if still present. The average monitoring period was 4.8 days (±0.249 s.e.; range: 4–8 days).

### Late parasitism

This was a follow-up to the “early parasitism”, conducted in the same nests. When natural clutch size reached 5 eggs, we exchanged one natural egg with a new conspecific egg, also painted yellow. We monitored nest contents for five consecutive days. The monitoring periods in early and late parasitism did not differ significantly (Mann–Whitney U_19,19_ = 123.5, p = 0.096).

### Late-only parasitism

We monitored the nest during laying and exchanged one host egg with a conspecific egg, painted yellow, on the day when the natural clutch sized reached 5 eggs. We checked these nests for 5 more consecutive days. We increased sample sizes (n = 12) by including previously collected data [32] from experimental parasitism with one late-stage, yellow parasitic egg (n = 12), as rejection rates proved to be similar (6/12 and 7/12 in the previous and present studies, respectively; χ^2^_1_ = 0.168, p = 0.682). We did not determine the age of the host in these previous experiments.

**Figure 3:**
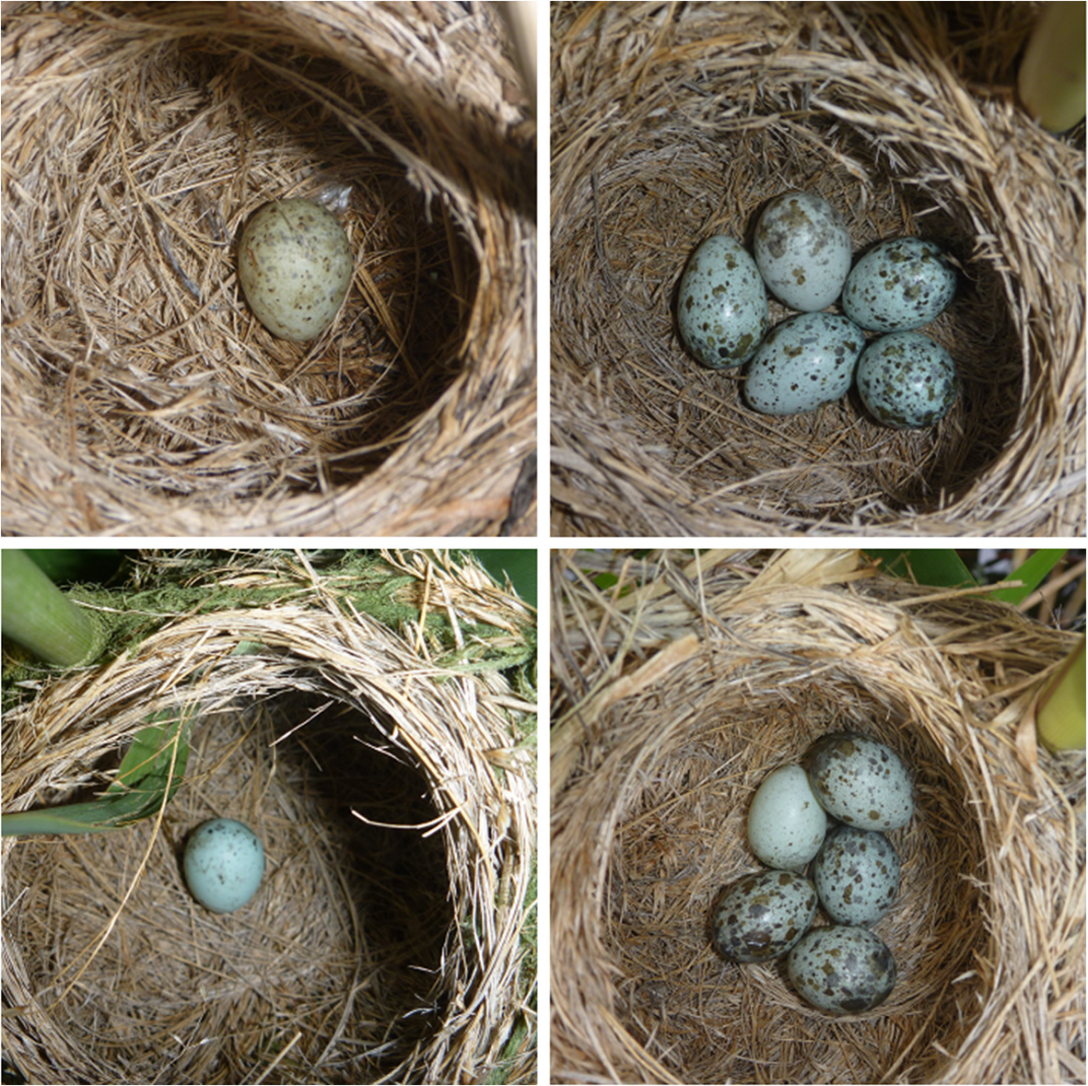
**Representative nests of great reed warblers with natural common cuckoo parasitism: two nests parasitized in the pre-egg laying stage (on the left), and two nests with cuckoo eggs in complete clutches (on the right).** In the latter nests the cuckoo egg is in the middle of the top positions (above), and in the left top position (below). (Photo credit: István Zsoldos).

Our experimental design followed the natural patterns of how cuckoos parasitize great reed warblers at our study site [29], with 10-15% of cuckoo eggs laid into still-empty nests preceding egg laying and similar frequencies of parasitism of clutches with five host eggs (Figure 3). Previous studies revealed that great reed warblers responded similarly to our experimental parasitic eggs as to real cuckoo eggs [31,32], and paint applied to the experimental eggs was not toxic for the embryos as shown by equal hatching rates between dyed and control eggs.

### Control

We monitored another set of host nests daily, without manipulation of clutch composition. We visited these nests daily and checked the content ensuring suitability as controls for detecting an observer effect (sensu [63]).

We categorized host responses to parasitism as acceptance, ejection of the parasitic egg, or desertion of the parasitized clutch (see [32] for more details). We detected three nest desertions in the “early parasitism” category, and two desertions in the “late parasitism” category. We did not consider desertion as rejection of parasitism in these analyses because desertion of nests with experimental yellow-dyed parasitic eggs is not typical in this host species [29]. Ejection cost, when a parasitic egg was ejected together with a host’s own egg, was observed in only one nest, in treatment “late parasitism”. No egg ejection or desertion was observed in the control nests (n *=* 27).

A linear mixed model was used to simultaneously evaluate which factors predicted host responses to experimental parasitism (dependent variable), including age (young/old), and timing of treatment (early/late) as factors, the interaction term age x timing, and nest identity as a random effect. We used StatXact version 10.0 for the calculation of confidence intervals for Spearman’s correlation and SPSS version 17 for all other statistical analyses.

## Competing interests

The authors declare that they have no competing interests.

## Authors’ contributions

CM, MB, and MEH designed the research; MB and CM performed the research; CM analyzed the data; and CM and MEH wrote the paper. All authors read and approved the final manuscript.
